# The Role of the Estrogen Pathway in the Tumor Microenvironment

**DOI:** 10.3390/ijms19020611

**Published:** 2018-02-19

**Authors:** Natalie J Rothenberger, Ashwin Somasundaram, Laura P. Stabile

**Affiliations:** 1 Department of Medicine, Division of Hematology/Oncology, University of Pittsburgh, Pittsburgh, PA 15232, USA; njr31@pitt.edu (N.J.R.); somasundarama@upmc.edu (A.S.); 2 Department of Immunology, University of Pittsburgh, Pittsburgh, PA 15213, USA; 3 Department of Pharmacology & Chemical Biology, University of Pittsburgh, Pittsburgh, PA 15213, USA; 4 UPMC Hillman Cancer Center, Pittsburgh, PA 15213, USA

**Keywords:** estrogen, cancer, tumor microenvironment, immunotherapy, immunosuppression

## Abstract

Estrogen receptors are broadly expressed in many cell types involved in the innate and adaptive immune responses, and differentially regulate the production of cytokines. While both genomic and non-genomic tumor cell promoting mechanisms of estrogen signaling are well characterized in multiple carcinomas including breast, ovarian, and lung, recent investigations have identified a potential immune regulatory role of estrogens in the tumor microenvironment. Tumor immune tolerance is a well-established mediator of oncogenesis, with increasing evidence indicating the importance of the immune response in tumor progression. Immune-based therapies such as antibodies that block checkpoint signals have emerged as exciting therapeutic approaches for cancer treatment, offering durable remissions and prolonged survival. However, only a subset of patients demonstrate clinical response to these agents, prompting efforts to elucidate additional immunosuppressive mechanisms within the tumor microenvironment. Evidence drawn from multiple cancer types, including carcinomas traditionally classified as non-immunogenic, implicate estrogen as a potential mediator of immunosuppression through modulation of protumor responses independent of direct activity on tumor cells. Herein, we review the interplay between estrogen and the tumor microenvironment and the clinical implications of endocrine therapy as a novel treatment strategy within immuno-oncology.

## 1. Introduction

Estrogens are pleiotropic steroids that play a regulatory role in a myriad of physiological processes from reproduction to lipid metabolism [1]. Biosynthetically converted from precursor androgens by the enzyme aromatase (CYP19A1), estrogens exert both genomic and non-genomic biological effects mediated by interactions with one of two cognate receptors, estrogen receptor α (ERα) or estrogen receptor β (ERβ). Albeit encoded by separate genes, both ER isoforms exhibit similar functional and structural organization [1]. Displaying high sequence homology within the DNA and ligand binding domains, both receptors interact similarly with endogenous estrogens, mainly 17β-estradiol (E2) [2,3]. In addition to mediating biological mechanisms involved in homeostasis, E2 also plays a role in the development and malignant progression of multiple cancers. The oncogenic role of estrogens is well characterized in both classical and nonclassical hormone-sensitive carcinomas including breast, prostate, endometrial, ovarian, colon, and lung [4]. ERs are located in both the nucleus and the cytoplasm of tumor cells enabling tumor-promoting transcriptional regulation of genes involved in cell survival and proliferation [5,6], and non-genomic crosstalk with growth factor pathways, including epidermal growth factor (EGF), insulin growth factor (IGF), and fibroblast growth factor (FGF) [7,8,9]. Due to these tumorigenic mechanisms, therapies that interfere with E2 signaling, such as selective estrogen receptor modulators or degraders (SERMs or SERDs) and aromatase inhibitors (AIs), have been developed and clinically implemented for the treatment of ER-positive breast cancer. While agents that target the estrogen pathway have been seminal in reducing breast cancer mortality over the past three decades [10], most studies in breast cancer and other cancer types have focused strictly on tumoral ER expression and signaling.

Along with tumor cells, non-cancerous cells comprising the tumor microenvironment (TME) are now recognized as critical mediators of tumor progression. Mounting evidence suggests that in addition to intracellular mechanisms such as mutational load and neoantigen presentation, interplay between cancer cells, stromal cells, immune cells, and extracellular molecules within the TME profoundly influence anti-tumor immunity and immunotherapeutic response [11,12,13,14]. The notion that enhancing tumor immunogenicity and inhibiting immunosuppressive mediators can functionally suppress progression of malignant tumors has led to the development of promising immunotherapeutic strategies. However, the clinical utility of current immunotherapies remains limited due to marginal response rates and acquired resistance mechanisms [15,16,17]. Therefore, greater elucidation of targetable cellular machinery involved in tumor immune evasion is necessary to improve the clinical benefit of immunotherapies.

The numerous biological effects of the E2 pathway are facilitated by distinct ER isoform expression found not only on tumor cells, but also on most immune cell types [18,19,20,21]. The impact of E2 in autoimmune pathogenesis remains heavily investigated, with reports of paradoxical and disease-dependent effects. The influence of E2 in autoimmunity is potentially concentration-dependent, and immune cell-specific. Several reviews detail E2-mediated immune responses, including transcriptional regulation of immune mediating genes possessing ERE sequences and regulation of lymphopoiesis and immune cell differentiation [22,23,24,25]. Given the prevalence of E2 modulation in both innate and adaptive immune responses, along with its evident role in tumor progression, there exist several implications for immunomodulatory effects of E2 within the TME. Herein, we will discuss findings within current literature evaluating the protumoral impact of E2 on the TME and the implications of targeting the E2 pathway in cancer to promote an anti-tumor immune response.

## 2. Estrogen Receptor and Aromatase Expression in Tumor Cells: Correlations with Clinical Outcome

Tumoral ER expression is reported in nearly 30 different types of cancer, predominately in hormone-sensitive tumors such as breast, ovarian, endometrial, and prostate [26,27]. Studies comparing clinicopathological characteristics with ER protein expression (typically evaluated by immunohistochemistry (IHC)) in tumor tissue show differential relation to disease prognosis based on cellular localization and cancer type. In breast cancer, while predominately expressed in the nucleus, ERα protein expression in either the nucleus and/or cytoplasm correlates with features of advanced disease, including larger tumor size and lymph node metastasis [28]. However, ERα-positive breast cancer patients exhibit improved overall survival (OS) compared to ERα-negative patients, likely owing to the clinical benefit of adjuvant endocrine therapies for ERα-positive patients [18,29]. The clinical relevance of ERβ expression in breast cancer remains controversial largely due to challenges associated with ERβ splice variants and post-translational modifications, as well as the lack of a clinically standardized ERβ antibody [19,30,31]. As an integral enzyme in estrogen production, intratumoral aromatase has also been evaluated in breast cancer. While one study reported an association between aromatase activity and poor prognosis, others have failed to correlate aromatase activity or protein expression with clinical outcomes, suggesting that paracrine sources of estrogen may be of greater significance in hormone-dependent breast cancers [32,33,34,35]. In contrast to breast cancer, non-small cell lung cancer (NSCLC) ERα protein expression is more commonly expressed in the cytoplasm and is a negative prognostic marker [36,37]. Similarly, elevated cytoplasmic ERβ protein expression in NSCLC is associated with poorer OS [38], potentially indicative of the predominance of non-genomic mechanisms in NSCLC. Alternatively, nuclear ERβ expression in NSCLC correlates favorably with OS in some studies and negatively in others (reviewed in [39]). Tumoral aromatase protein expression and activity is also reported in NSCLC, with elevated expression identified as a predictor of poorer survival in women with early stage disease [40]. In advanced ovarian cancer tumors, while aromatase activity and ERβ mRNA expression do not correlate with any clinical outcomes [41,42], a recent meta-analysis revealed ERα protein expression was associated with improved OS [43]. Finally, while clinical correlations with aromatase have yet to be evaluated, both ERα and ERβ expression are associated with improved OS in endometrial cancer [44]. These clinical correlations, combined with mounting preclinical studies, indicate an intricate and pervasive protumoral role for hormonal signaling in multiple cancers, providing rationale for further investigation of ER expression and oncogenic cellular modulation.

## 3. Estrogen Receptor and Aromatase Expression and Estrogen-Mediated Effects in the Tumor Microenvironment

In addition to neoplastic cells, ERs and aromatase are also expressed on stromal and immune cells within the TME (Table 1). Numerous studies over the past decade have demonstrated that interactions between tumor cells and surrounding recruited stromal cells are integral in disrupting homeostasis and potentiating tumorigenesis (reviewed in [14,45]). Albeit highly heterogeneous within and across tumor types, regularly observed cellular components of the TME include: cancer associated fibroblasts (CAFs), tumor associated macrophages (TAMs), myeloid derived suppressor cells (MDSCs), immune T and B cells, natural killer (NK) cells, and endothelial cells [14]. ER and aromatase expression in TME stromal and immune cells suggest a potential immunomodulatory role of ER signaling in cancer biology as detailed by cell type below.

### 3.1. Stromal Cells

It has become increasingly evident that tumor progression is reliant not only on tumor cells present in malignant tissue, but also the distinctive stromal cells recruited to the TME that signal among the tumor cells and each other. An in vivo murine model evaluating tumor cell-independent mechanisms of ER signaling within the TME has identified ERα expression and modulation in stromal cell types. In ovariectomized syngeneic mice transplanted with ER-negative melanoma, breast, or lung cancer cells, E2 treatment significantly enhanced tumor growth of each cell type compared to untreated controls via interactions with stromal ERα [47]. Further, E2-stimulated tumor growth was increased when evaluated in immunocompromised mice, suggesting this effect may be more reliant on the innate immune response [47]. In addition to tumor growth, E2 also enhanced angiogenesis by increasing blood vessel density 2.1-fold in E2-treated mice compared to controls, an effect reliant on host ERα expression [47]. Peritumoral aromatase expression is also reported in endometrial cancer stromal cells, correlating with advanced disease and poor OS [48,58]. Aromatase is also observed in breast cancer stromal adipocytes of obese postmenopausal women, and several studies have identified mechanistic associations between obesity, inflammation, elevated aromatase, and breast cancer development [46,59,60].

### 3.2. Cancer Associated Fibroblasts

CAFs are among the most prevalent stromal cell type within the TME and act as a paracrine source of chemokines and soluble growth factors that activate signaling pathways involved in tumor cell survival, invasion, and metastasis [61]. A study using nuclear receptor arrays to compare gene expression profiles between normal human breast adipose fibroblasts and primary CAFs from malignant human breast tissue, observed ERα expression in fibroblasts from primary breast cancer tissue [49]. Despite similar levels of ERα expression observed in both cancerous and normal fibroblasts, the E2 responsive gene, liver receptor homolog-1 (*LRH-1*) was upregulated in CAFs compared to normal fibroblasts [49]. *LRH-1* is also an estrogen response gene and a direct transcriptional regulator of the aromatase encoding gene *CYP19A1* [62,63,64]. Aromatase is found to be co-expressed in breast cancers with LRH-1, suggesting a paracrine mechanism of E2 synthesis and ER-mediated oncogenesis in the breast cancer TME [65]. Endometrial CAFs also express both ERs and can promote tumor cell proliferation when co-cultured with human endometrial tumor cells [52]. Endometrial CAFs induce in vitro tumor cell proliferation in part through activation of the phosphatidylinositide 3-kinase (PI3K) and mitogen-activated protein kinase (MAPK) signaling networks, which are well-known ER-mediated pathways in breast and lung cancer [52,66,67,68].

ERα is also expressed in prostate CAFs, however, clinical implications remain unclear with some reports identifying CAF ERα and ERβ expression as a marker of clinically advanced disease [50], while other reports suggest ERα expressing CAFs provide a protective effect against tumor cell invasion and macrophage infiltration [69,70]. In the latter studies, stromal ERα reduced both murine and human prostate cancer cell invasion using an in vitro co-culture system, and reduced lymph node metastasis of orthotopically implanted human prostate cancer cells in mice [70]. Mechanistically, ERα-positive CAFs abated migratory behavior of adjacent prostate tumor cells through reduced expression of C–C motif chemokine ligand 5 (CCL5) and IL-6 chemokines, both of which have identified roles in tumor immune recruitment, inflammation, and activation of growth factor signaling [71,72].

### 3.3. Tumor Associated Macrophages

Macrophages critically regulate innate immune responses under normal physiological conditions; however, several studies have shown that TAMs can promote tumor cell proliferation, an inflammatory microenvironment, and metastasis [73,74]. Macrophage immune responses are tissue-specific and dependent on polarization by different cytokines within the local microenvironment [75]. Fully polarized M1 macrophages produce proinflammatory cytokines including IFNγ, interleukin 12 (IL-12), and TNFα, that contribute to tumor rejection and antigen presentation [75]. Alternatively, macrophages exhibiting an M2 phenotype produce type-2 cytokines including interleukins 4,5,6, and 10 [75], all of which are identified promoters of tumor progression through enhanced tumor cell growth and immune evasion [76]. Infiltrating TAMs observed in malignant tumors display an M2 phenotype, representing another potential protumoral therapeutic target within the TME. TAM infiltration is observed in a wide-range of cancer types and correlates with poor prognosis [77]. For example, TAM infiltration is an independent poor prognostic predictor for ovarian cancer, with higher infiltration observed in cancerous specimens compared to benign lesions, and density-dependent associations with five-year survival rates [78].

Co-localized expression of both ERα and ERβ is reported in human high grade serous ovarian cancer (HGSOC) TAMs, and premenopausal patients show elevated TAM infiltration compared to postmenopausal women, with highest overall TAM density observed in ERα-positive tumors [54]. Conversely, while TAM infiltration has been associated with poor prognosis in both hormone receptor positive and negative breast cancers, TAM enrichment and proliferation is more commonly observed in hormone receptor negative breast tumors [79,80]. However, M1 versus M2 polarization was not evaluated in these studies. Furthermore, a separate IHC analysis of breast cancer specimens revealed aromatase expression in TAMs, enabling local E2 production within the TME and enhanced ER-positive breast tumor cell proliferation [55]. Aromatase is also expressed in TAMs from NSCLC patient tumors [56], and both aromatase and ERβ are observed in infiltrating macrophages of preneoplasias in tobacco carcinogen-induced murine lung tumors [57].

While a paucity of data exists regarding ER expression in TAMs of several cancer types, there is evidence that E2 can induce M2 polarization and tumor infiltration. Using a polyomavirus middle T (PyMT) ER-positive breast cancer murine model, E2 increased tumoral M2 TAM infiltration, while untreated controls alternatively exhibited M1 TAM infiltration [81]. Furthermore, E2 enhanced M2 macrophage secretion of vascular endothelial growth factor (VEGF), an identified mediator of M2 macrophage recruitment [81,82]. E2 has been shown to also upregulate VEGF expression and pulmonary macrophage content in the lungs of mice exposed to a tobacco carcinogen [83]. Evaluation of E2-mediated tumor growth in a HGSOC murine model showed that E2 not only enhanced the growth of ER-negative xenografts, but also increased M2 TAM infiltration compared to untreated ovariectomized mice [54]. In addition to reports of E2-mediated TAM infiltration, a tissue microarray of patient samples coupled with in vitro analysis revealed endometrial M2 TAMs mediate ER activation through epigenetic upregulation of ERα by secreted interleukin-17A (IL-17A), increasing E2-driven malignant endometrial cell proliferation [84]. Taken together, these studies suggest a potential positive feedback mechanism between the estrogen pathway and M2 TAM infiltration in certain cancers. Targeting this interaction may therefore provide therapeutic benefit as recently demonstrated in a lung cancer xenograft model using the phytoestrogen SERM resveratrol [85]. The study showed resveratrol treatment significantly suppressed tumor growth by inhibiting M2 polarization of TAMs and decreasing activation of signal transducer and activator of transcription 3 (STAT3) signaling [85].

### 3.4. Myeloid Derived Supressor Cells

MDSCs are another myeloid cell present in the TME known to disrupt immune surveillance and promote tumor development [86]. ERα expression was also recently identified by IHC and confirmed by PCR and immunoblotting in MDSCs isolated from the tumor, bone marrow, and peripheral blood of human ovarian cancer patients [53]. Using an E2-insensitive syngeneic ovarian cancer model, ovariectomized mice exhibited improved survival compared to non-ovariectomized mice following tumor challenge, while E2 supplementation accelerated tumor progression and reversed the protective effect found in estrogen-depleted mice [53]. Notably, this effect was only observed in immunocompetent mice with no survival benefit of ovariectomy observed in tumor-bearing T-cell deficient immunocompromised mice, suggesting the antitumor effects of E2 deficiency is reliant on functional adaptive immunity [53]. E2-treated mice also exhibited significantly fewer helper and cytotoxic T cells, but also exhibited significantly elevated recruitment of MDSCs in both the spleen and tumor beds [53]. Specifically, the immunosuppressive activity of granulocytic MDSCs was increased in this model. ER-dependence of MDSC expansion was demonstrated using the ERα antagonist methylpiperidino pyrazole (MPP) to inhibit MDSC proliferation in vitro [53]. In the peritoneal cavity of ovarian tumor-bearing mice, E2 treatment increased activation of STAT3 signaling, a regulator of myeloid differentiation and development [87], through transcriptional upregulation of JAK2 and SRC activity [53]. Similar findings were also observed in syngeneic lung and breast cancer murine models and the E2-stimulated tumor growth was abrogated by MDSC depletion using anti-Gr1 antibodies [53].

### 3.5. Tumor Infiltrating Lymphocytes (TIL)

Lymphocyte composition of the TME vastly differs based on cancer type and immune infiltrates exhibit opposing properties promoting tumor progression and antitumor immunity depending on the primary tumor [88]. For example, CD4^+^ T cell polarization has been identified as a mediator of tumor immune surveillance. T helper 1 (Th1) T cell responses are associated with tumor suppression and upregulation of IFNγ and IL-12, while T helper 2 (Th2) responses are reliant of IL-4 production and exhibit protumor activity [89,90]. Interestingly, several murine and human studies report elevated E2 induces increased Th2 responses and upregulate IL-4 production [22,25]. A recent study utilizing an in silico machine learning based approach, identified increased immune infiltrate including Th1 T cells, B cells, and cytotoxic T lymphocytes (CTLs) in ER-negative breast tumors relative to ER-positive breast tumors [91]. This study observed an inverse correlation between ER activity and immune infiltration of each of these cells in breast cancer tissues, confirming previous reports that increased TIL, specifically CD8^+^ T cells, in ER-negative tumors significantly correlates with improved OS [91,92]. Furthermore, a post-hoc analysis of gene expression in ER-positive breast cancer patients showed that treatment with the AI letrozole increased the infiltration of B cell and T helper lymphocyte subsets at early and late time points following treatment initiation [91].

#### 3.5.1. Cytotoxic T Cells and Natural Killer Cells

Granule-mediated exocytosis is one pathway by which CTLs and NK cells initiate apoptosis to eliminate pathogenic and tumor cells [93]. Serine proteases such as granzyme B are deposited into the target cells to initiate caspase-dependent apoptosis [94]. Jiang et.al. cultured ERα expressing human liver carcinoma cells with E2 and showed E2 treatment upregulated expression of the granzyme B inhibitor, proteinase inhibitor-9 (PI-9), and protected the cells against NK and CTL-induced apoptosis in DNA fragmentation assays [95]. E2-induced PI-9 expression was also observed in ERα-positive MCF7 breast cancer cells, again protecting cells against NK elimination, while PI-9 knockdown blocked E2’s protective effect against NK granule-mediated apoptosis [96]. These studies suggest that E2 enhances immunosuppression through inhibition of NK and CTL-mediated tumor cell elimination.

#### 3.5.2 Regulatory T Cells

T cell activation and effector differentiation is an essential part of the adaptive immune response. FoxP3 expressing Tregs are integral in coordinating suppression of anti-tumor immune responses, secreting immunosuppressive cytokines and inhibiting responder T cell expansion [97]. Physiological doses of E2 administered to immunocompetent ovariectomized female mice have been shown to enhance CD4^+^CD25^+^ Treg expansion and upregulate Foxp3 expression in multiple tissues [98]. Furthermore, fluorescence-activated cell sorting (FACs) assays revealed ERα expressing CD4^+^CD25^-^ cells incubated with E2 acquire CD25 expression [98]. E2 transformed CD4^+^CD25^+^ T cells exhibited an immunosuppressive Treg phenotype, significantly inhibiting T cell proliferation in an in vitro mixed lymphocyte reaction [98]. Additional studies have reported E2-stimulated Foxp3 expression in murine Tregs, which is of importance considering that Foxp3 is essential for Treg functionality, and tumoral aggregation of FoxP3^+^ Tregs in patients is a predictor of poor prognosis in multiple cancers [99,100,101]. For example, in early-stage NSCLC patients, nuclear ERα expression was found independently associated with increased risk of recurrence and FoxP3^+^ lymphocyte infiltrate [102]. Further, a recent meta-analysis reported FoxP3^+^ Treg infiltration significantly correlated with poorer OS in ER-positive breast cancer patients, but improved survival rates in ER-negative patients [103]. In addition, evaluation of ERα-positive breast tumors from patients treated with letrozole showed a significant reduction of FoxP3^+^ Tregs post-treatment [104].

Moreover, Tregs isolated from mice treated with E2 displayed enhanced suppression and increased intracellular expression of the immune checkpoint protein programmed death-1 (PD-1), while ERα and ERβ knockout reduced Treg suppression and PD-1 expression [105]. Of note, E2 treatment also stimulates in vitro expression of the PD-1 ligand (PD-L1) on ERα-positive endometrial and breast cancer cells through activation of PI3K signaling [106]. Interactions between PD-L1 expressing tumor cells and PD-1 positive T cells induces cytotoxic T cell exhaustion, resulting in tumor immune evasion [107]. Evidence that E2 upregulates both PD-L1 and PD-1, suggests E2 signaling may critically influence the PD-1/PD-L1 pathway.

### 3.6. Inflammatory Cytokines and Eicosanoids

Chronic inflammation is widely recognized as an ancillary mechanism promoting tumor progression. The TME releases cytokines that activate protumoral pathways mediating proliferation, immune evasion, and metastasis [108]. IL-6, a proinflammatory cytokine, has been shown to enhance ERα-positive breast cancer cell growth and invasion [109]. Local TAFs in breast cancers act as a paracrine source of the elevated IL-6, driving STAT3 activation and ERα-positive tumor cell proliferation both in vitro and in vivo [110]. TNFα, another ubiquitous TME cytokine, regulates expression of genes associated with metastatic phenotypes in ERα-positive breast cancer cells [111]. TNFα has also been shown to upregulate aromatase expression in cultured human adipose stromal cells [112]. Transcriptional linear correlations between aromatase and the cytokines TNFα and IL-6 have been reported in patient breast cancer tissue, but not in adjacent non-cancerous tissue [113]. A similar correlation has also been seen between aromatase and the eicosanoid cyclooxygenase-2 (COX-2) [113]. COX-2 is responsible for the synthesis of inflammatory promoting eicosanoids such as prostaglandin E2 (PGE2) [114]. It is well established that PGE2 promotes upregulated transcription of aromatase through elevated cyclic adenosine monophosphate (cAMP) in breast tumors [115]. Despite conflicting reports, some epidemiological studies show that regular use of COX-2 inhibiting nonsteroidal anti-inflammatory drugs (NSAIDs) can reduce the risk of developing ERα-positive breast cancers, but not ERα-negative cancers [116].

Significant correlations between ERα, TNFα, and NF-κB protein expression have also been reported in breast cancer tissues [117]. NF-κB signaling is well recognized for its role in tumor initiation and inflammation [118]. Constitutive activation of NF-κB is observed in several cancers, and is associated with the cytokines IL-6 and TNFα [118]. Increased DNA binding of NF-κB and activator protein-1 (AP-1) has been observed in SERM-resistant, ERα-positive breast cancer cell line models and patient specimens [119,120]. Furthermore, E2 exposure in a murine model evaluating tobacco-induced lung cancer enhanced pulmonary inflammation through increased activation of NF-κB signaling and expression of VEGF and IL-17A [83]. Alternatively, targeting E2 and inflammatory pathways with combined AI and NSAID treatment maximally prevented carcinogen-induced lung tumor development in mice, significantly reducing STAT3 and MAPK signaling, circulating IL-6, and IL-17A expression [83]. Taken together, these reports indicate potential interactions between the E2 pathway and regulators of tumor-promoting inflammation, representing another beneficial target of E2 inhibition.

## 4. Clinical Implications of Targeting the Estrogen Pathway in the Tumor Microenvironment

Immunotherapy is a powerful therapeutic strategy for cancer; however, the immunosuppressive TME poses major obstacles for this approach. Currently, immune checkpoint inhibitors of cytotoxic T-lymphocyte-associated antigen 4 (CTLA4) and PD-1/PD-L1 are among the most clinically evaluated immune therapies [121]. These agentshave remarkably advanced cancer treatment, significantly improving response rates and survival compared with standard-of-care chemotherapies [122,123,124,125]. However, typical response rates to these therapies remain limited to only around 20–35% of patients, with variable responses depending on stage, tumor type, and PD-L1 staining positivity [126]. Furthermore, while some patients have durable responses, mechanisms of acquired and adaptive resistance are becoming apparent, with 25 to 33% of melanoma patients exhibiting delayed relapse on these therapies [15,16].

**Figure 1:**
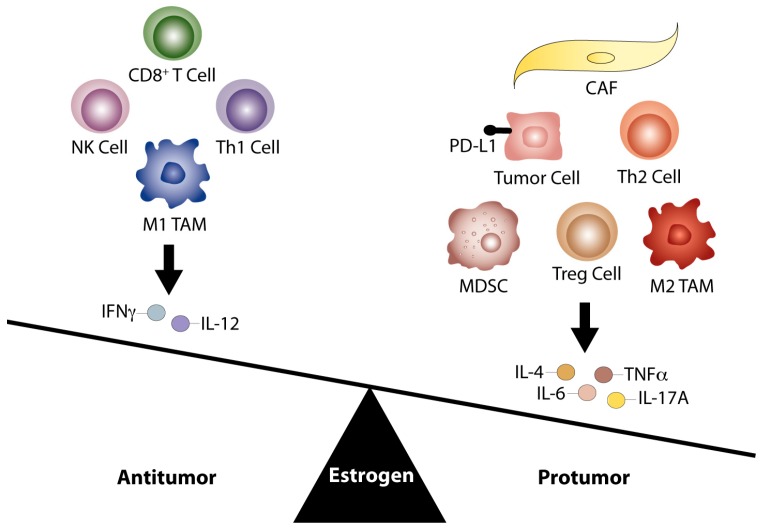
The E2 pathway promotes a protumor TME. The E2 pathway contributes to aberrant regulation of antitumor immunity, enhancing a greater number of protumoral responses within the TME. Current literature suggests E2 may facilitate an immunosuppressive TME by shifting the balance in favor of Th2 responses, production of tumor-promoting cytokines (IL-6, IL-4, TNFα, and IL-17A), and M2 TAM infiltration compared to Th1 responses, associated Th1 cytokines (IL-12 and IFNγ), and M1 TAM infiltration. E2 may further promote tumor immune evasion through proliferation of Treg and MDSC populations, increased tumor cell PD-L1 expression, and inhibition of CD8^+^ T cell and NK cell induced apoptosis. CAFs may additionally support a protumor environment by supplying paracrine sources of E2 and IL-6. Therefore, targeted inhibition of the E2 pathway may act as a novel strategy to enhance the effects of immunotherapies and reverse this immune imbalance within the TME.

Recent efforts to identify molecular events underlying immune evasion and failed therapeutic response report that damaged DNA repair mechanisms, increased non-synonymous somatic mutational load, and neoantigen presentation correlate with tumor immunogenicity and improved clinical outcomes [12,13,127]. Alternatively, mechanisms facilitating immune evasion involve damage to antigen presenting capacity and recurrence of non-antigenic mutations poorly presented by MHC class 1 molecules [128,129]. While these findings provide a greater understanding of tumor immunoediting and potential biomarkers predictive of response, novel therapeutic combinations are still needed to improve the efficacy of current immunotherapeutic agents. The identification of E2 modulation of the tumor immune phenotype justifies investigation of endocrine agents to reverse tumor immune tolerance. As depicted in Figure 1, E2 signaling can modulate the immune TME through enhanced protumoral responses. Therefore, anti-estrogen therapy has the potential to not only reverse an immunosuppressive TME, but also to augment response in E2-sensitive tumors.

Recently, a high-throughput screening assay in lung cancer cells identified the anti-estrogen fulvestrant as the top compound that increased tumor sensitivity to immune-mediated lysis [130]. Fulvestrant is an ideal candidate to combine with anti-PD-1/PD-L1 agents, due to its proven safety profile and non-overlapping toxicities. These new findings of E2 action on immune cells could create a paradigm shift towards utilizing anti-estrogen therapy to target the immunosuppressive TME, thereby increasing the efficacy and duration of response of current immunotherapies [131].

## 5. Conclusions and Perspective

The E2 pathway is an identified promoter of tumorigenesis in several cancers, largely for its direct genomic and non-genomic effects on tumor cells. However, evidence of ER and aromatase expression on stromal and immune cells within the TME indicates that additional mechanisms exist by which estrogens enhance malignant progression. It is becoming increasingly evident that cells comprising the TME can impact tumor immunity, either beneficially through enhanced antitumoral immune responses, or detrimentally through increased protumoral responses. Evidence thus far suggests that E2 facilitates a primarily tumor-promoting and immunosuppressive TME in multiple tumor types. While checkpoint blockade immunotherapies have exhibited significant clinical success for the treatment of certain cancers, partial response rates and acquired resistance to these therapies necessitate the development of strategies to boost immunotherapeutic responses. The data summarized here points to the E2 pathway as a regulator of tumor immune responses, suggesting that clinical benefit may be derived from combining estrogen blocking agents with immune checkpoint inhibitors. Prior to clinical analysis of this combination, a more comprehensive characterization of E2-related proteins in the TME of various tumor types is necessary. There is also a need for standardized methods and CLIA-approved assays for the detection of ERβ and aromatase expression. Future studies evaluating response to current immunotherapies based on sex-differences, patient demographics including menopausal status, and obesity are warranted, given the pervasive involvement of the E2 pathway in tumor immunity.

## Figures and Tables

**Table 1:** Estrogen receptor (ER) and aromatase expression in stromal and immune cells in the tumor microenvironment.

TME Cell Type	Cancer Type	Human Expression	Murine Expression	Method of Evaluation	Reference
Stromal	Breast	Aromatase	ERα	PCR, IHC	[46,47]
	Melanoma		ERα	IHC	[47]
	Lung		ERα	IHC	[47]
	Endometrial	Aromatase		IHC	[48]
CAF	Breast	ERα		PCR	[49]
	Prostate	ERα, ERβ		IHC	[50,51]
	Endometrial	ERα, ERβ		PCR	[52]
	Ovarian	ERα		IHC	[53]
TAM	Ovarian	ERα, ERβ		IF, IHC	[54]
	Breast	Aromatase		IHC, PCR	[55]
	Lung	Aromatase	Aromatase	IHC	[56,57]
MDSC	Ovarian	ERα	ERα	PCR, Western	[53]

Studies were identified by PubMed searches using keywords: ERα, ERβ, aromatase, stromal, CAF, TAM, MDSC, expression, cancer. CAF: cancer associated fibroblast; TAM: tumor associated macrophage; MDSC: myeloid derived suppressor cell; IHC: immunohistochemistry; PCR: polymerase chain reaction; IF: immunofluorescence; Western: western blotting analysis.

## Abbreviations

ERβ	Estrogen receptor β
ERα	Estrogen receptor α
ERE	Estrogen response element
E2	17β-Estradiol
DC	Dendritic cell
Treg	Regulatory T cell
SERM	Selective estrogen receptor modulator
SERD	Selective estrogen receptor degrader
IHC	Immunohistochemistry
IF	Immunofluorescence
Th2	T helper 2
Th1	T helper 1
IL-4	Interleukin-4
IFNγ	Interferon Gamma
IL-6	Interleukin-6
TNFα	Tumor necrosis factor alpha
TME	Tumor microenvironment
EGF	Epidermal growth factor
IGF	Insulin growth factor
FGF	Fibroblast growth factor
OS	Overall survival
NSCLC	Non-small cell lung cancer
CAF	Cancer associated fibroblast
TAM	Tumor associated macrophage
MDSC	Myeloid derived suppressor cell
MPP	Methylpiperidino pyrazole
NK	Natural killer
LRH-1	Liver receptor homolog-1
PCR	Polymerase chain reaction
PI3K	Phosphatidylinositide 3-kinase
MAPK	Mitogen-activated protein kinase
CCL5	C–C motif chemokine ligand 5
IL-12	Interleukin-12
VEGF	Vascular endothelial growth factor
HGSOC	High grade serous ovarian cancer
IL-17A	Interleukin-17A
STAT3	Signal transducer and activator of transcription 3
TIL	Tumor infiltrating lymphocyte
AI	Aromatase inhibitor
CTL	Cytotoxic T lymphocyte
PI-9	Proteinase inhibitor-9
PD-1	Programmed death-1
PD-L1	PD-1 ligand
COX-2	Cyclooxygenase-2
PGE2	Prostaglandin E2
NSAID	Nonsteroidal anti-inflammatory drug
AP-1	Activator protein-1
CTLA4	Cytotoxic T-lymphocyte–associated antigen 4
